# Identification of novel viral receptors with cell line expressing viral receptor-binding protein

**DOI:** 10.1038/srep07935

**Published:** 2015-01-21

**Authors:** Mei Mei, Jianqiang Ye, Aijian Qin, Lin Wang, Xuming Hu, Kun Qian, Hongxia Shao

**Affiliations:** 1 Ministry of Education Key Lab for Avian Preventive Medicine, Yangzhou University, No.12 East Wenhui Road, Yangzhou, Jiangsu. 225009, P. R. China; 2 Key Laboratory of Jiangsu Preventive Veterinary Medicine, Yangzhou University, Yangzhou. 225009, P. R. China; 3 Jiangsu Co-innovation Center for Prevention and Control of Important Animal Infectious Diseases and Zoonoses, Yangzhou. 225009, P. R. China

## Abstract

The viral cell receptors and infection can be blocked by the expression of the viral receptor-binding protein. Thus, the viral cell receptor is an attractive target for anti-viral strategies, and the identification of viral cell receptor is critical for better understanding and controlling viral disease. As a model system for viral entry and anti-retroviral approaches, avian sarcoma/leukosis virus (ASLV, including the A-J ten subgroups) has been studied intensively and many milestone discoveries have been achieved based on work with ASLV. Here, we used a DF1 cell line expressed viral receptor-binding protein to efficiently identify chicken Annexin A2 (chANXA2) as a novel receptor for retrovirus ALV-J (avian leukosis virus subgroup J). Our data demonstrate that antibodies or siRNA to chANXA2 significantly inhibited ALV-J infection and replication, and over-expression of chANXA2 permitted the entry of ALV-J into its non-permissible cells. Our findings have not only identified chANXA2 as a novel biomarker for anti-ALV-J, but also demonstrated that cell lines with the expression of viral receptor-binding protein could be as efficient tools for isolating functional receptors to identify novel anti-viral targets.

The binding of the viral surface protein to the receptors expressed in host cells triggers the viral infection and pathogenesis^1^,^2^,^3^. Thus, viral cell receptors not only determine the viral tropism, but also provide host targets for antiviral strategies. For example, the multiple identified cell receptors and co-receptors for HIV (e.g., CD4, CCR5, and CXCR4) are clarifying the molecular details of HIV entry and creating efficient approaches for AIDS interventions^4^,^5^,^6^,^7^. And the sialic acid analogues that mimic the influenza virus receptors have been shown clinical effects against influenza infection^8^. The receptor for SARS coronavirus (SARS-CoV), angiotensin-converting enzyme 2, has also been reported as a potential therapeutic target for SARS-CoV^9^,^10^.

As a model system for viral entry, avian sarcoma/leukosis virus (ASLV, including A–J ten subgroups) has been studied intensively, and several important receptors for ASLV entry have been identified by traditional methods^11^,^12^,^13^,^14^,^15^. Because saturation of the viral cell receptors of susceptible cells via the expression of viral receptor-binding protein can block the corresponding viral infection^16^,^17^,^18^, such virus-resistant cells might be efficient tools for the isolation of the functional receptors for viral entry and novel anti-viral biomarkers. To test this possibility, we used an ALV-J-resistant cell line (pcDNA-env_DF1) that expresses ALV-J Env in the ALV-J-susceptible cell line DF1 as a tool for isolating novel receptors for ALV-J. Through this approach, we identified chicken Annexin A2 (chANXA2) as a novel ALV-J receptor.

## Results

### Identification of chANXA2 as a novel binding protein to ALV-J Env

**Figure 1:**
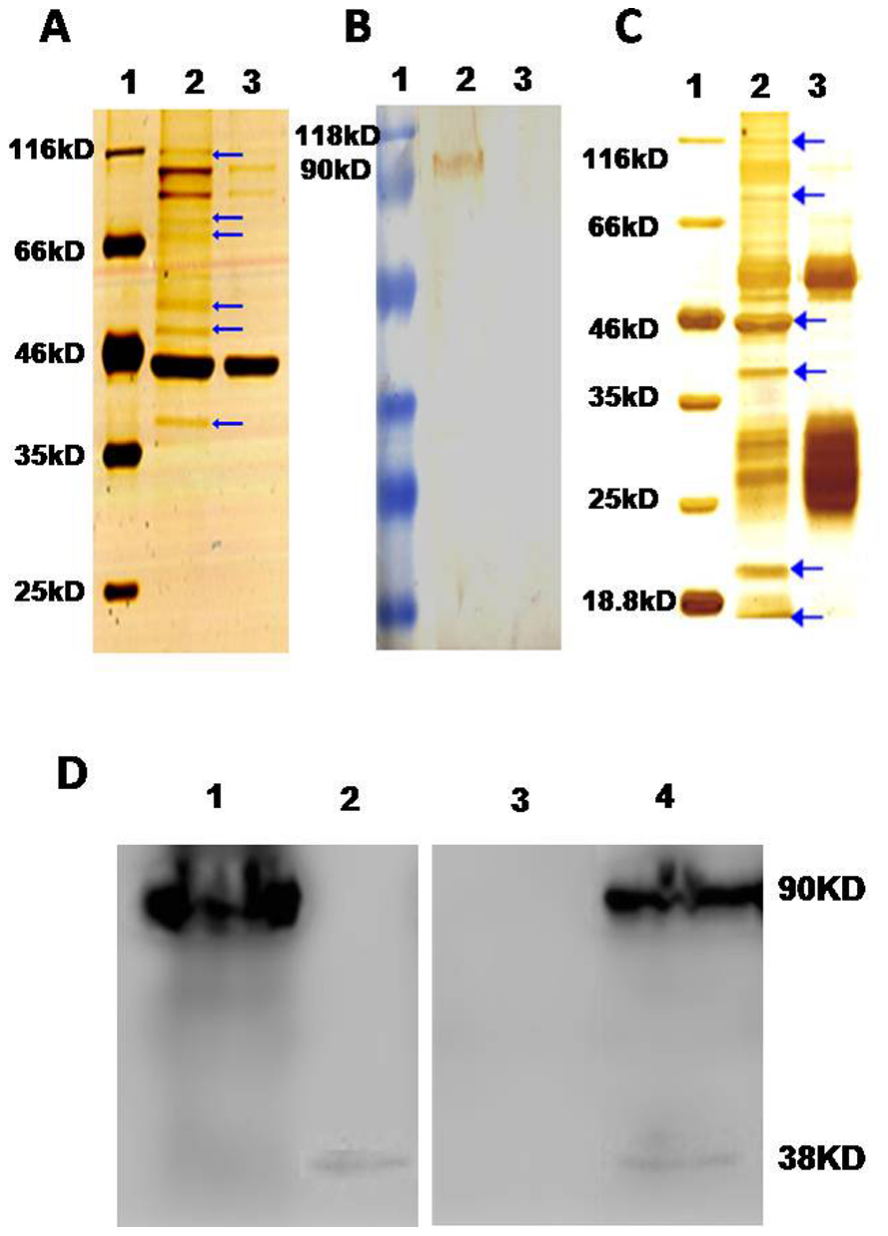
(Qin) chANXA2 binding to ALV-J Env protein (A), Silver Staining of protein precipitation for the membrane proteins of the pcDNA-env_DF1 cells. Lane 1, protein marker; lane 2, precipitated with JE9; lane 3, precipitated with isotype control IgG. (B), The fusion protein SUJ-rIgGFc was analyzed by western blotting with JE9. Lane1, protein marker; lane2, lysate of MDCK cells infected with rAd-SUJ-rIgGFc; lane3, lysate of MDCK cells infected with wild type rAd; (C), Silver Staining of protein precipitation for the membrane proteins of DF1 cells. Lane 1, protein marker; lane 2, precipitated with SUJ-rIgGFc; lane 3, precipitated with rabbit IgG. (D), Western blot assay for the co-immunoprecipitation. Lane 1, 293T cell transfected with pcDNA3.1-EnvJ were analyzed with JE9; lane 2, 293T cell transfected with chANXA2 were analysed with anti-chANXA2 (C-16); lane 3, 293T cell lysates transfected with chANXA2 immunoprecipitated with JE9 and analyzed with anti-chANXA2 (C-16); lane 4, 293T cell co-transfected with pcDNA3.1-EnvJ and chANXA2 were immunoprecipitated with JE9, and analyzed with JE9 and anti-chANXA2 (C-16).

The pcDNA-env_DF1 cell line expressing ALV-J Env protein was previously constructed and shown to be resistant to ALV-J infection^18^. To use this cell line to isolate novel functional receptors for ALV-J, we first extracted the membrane proteins from the pcDNA-env_DF1 cells and then performed immunoprecipitation with the single monoclonal antibody (mAb) JE-9, which is specific to ALV-J Env^19^. Silver staining for SDS-PAGE of the immunoprecipitation revealed several different bands in the lysate that was immunoprecipitated with ALV-J-specific mAb JE-9 and not with the control antibody (Fig. 1A). Mass spectrometry further revealed that one of these bands was chicken Annexin A2 (chANXA2), a member of the annexin family^20^.

To further confirm this finding, a recombinant adenovirus rAd-SUJ-rIgGFc expressing fusion protein SUJ-rIgGFc (Fig. 1B) was constructed and the purified SUJ-rIgGFc was used to precipitate the membrane protein extracted from DF1 cells. SDS-PAGE and Mass spectrometry (MS) revealed that chANXA2 was also found in the precipitate with purified SUJ-rIgGFc, but not in the precipitate with rabbit IgG control protein (Fig. 1C). Moreover, we cloned the full-length cDNA encoding chANXA2 from the total RNA of the DF1 cells into the pcDNA3.1 vector, and did co-transfection with plasmid pcDNA3.1_EnvJ and chANXA2 in 293T cells. The co-immunoprecipitation (co-IP) using mAb JE9 revealed that ALV-J Env protein could efficiently interact with chANXA2 (Fig. 1D). All these data clearly demonstrate that chANXA2 is identified as a novel binding protein to ALV-J Env.

### Antibody or siRNA to chANXA2 significantly inhibiting ALV-J infection and replication

**Figure 2:**
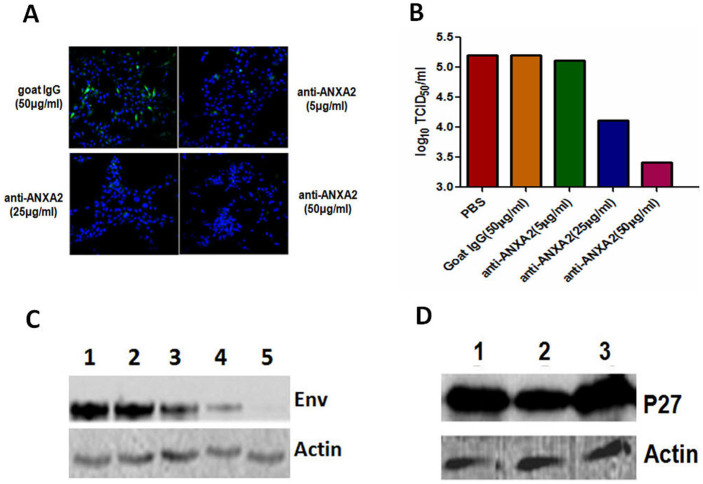
(Qin) Inhibition of ALV-J infection by antibodies to ANXA2. The DF1 cells that had been pre-treated with antibodies against ANXA2 were infected with ALV-J or ALV-A, and the replications of ALV-J and ALV-A in the treated cells were analysed. (A), IFA analysis using JE9; (B), TCID_50_ analysis for viral titres; (C), western blot analysis for the expression of Env from ALV-J in the DF1 cells that were treated with antibodies. Lane1, mock; lane 2, goat IgG (50 μg/ml); lane 3, anti-ANXA2 (5 μg/ml); lane 4, anti-ANXA2 (25 μg/ml); lane 5, anti-ANXA2 (50 μg/ml); (D), western blot analysis for the expression of p27 of ALV-A in the DF1 cells treated with antibodies. Lane 1, mock; lane2, goat IgG (50 μg/ml); lane 3, anti-ANXA2 (50 μg/ml).

To test whether chANXA2 serves as a functional receptor for ALV-J infection, we used antibodies against chANXA2 to perform blocking assays to evaluate the effects of chANXA2 on ALV-J infection in DF1 cells. Our results revealed that the viral infection/replication of ALV-J was significantly inhibited in groups that had been treated with anti-ANXA2. As shown in Fig. 2A, there was little visible immunofluorescence in the cells that were treated with 50 or 25 μg/ml of the antibody against chANXA2 in the IFA. Moreover, only a few positive cells were found among the cells that were treated with 5 μg/ml of antibody against ANXA2. In contrast, many positive cells were found among the cells that were treated with the control IgG and among the untreated cells. Consistent with the IFA results, the viral titres of the cells that were treated with 50 and 25 μg/ml of antibody against chANXA2 were approximately 50-fold and 10-fold less, respectively, than those of the cells that were treated with the control IgG (Fig. 2B). The inhibitory effect on ALV-J infection/replication conferred by the antibody against ANXA2 was also confirmed by western blot (Fig. 2C). As a viral control, we also performed a blocking assay for ALV-A infection in the DF1 cells. As described in Fig. 2D, the p27 expression levels of ALV-A in the groups that were treated with the antibody against chANXA2 were similar to those of the mock group, which indicates that the antibody against ANXA2 could not inhibit ALV-A infection/replication in DF1 cells. These data clearly demonstrate that blocking chANXA2 with a specific antibody can effectively and specially inhibit the infection/replication of ALV-J.

**Figure 3:**
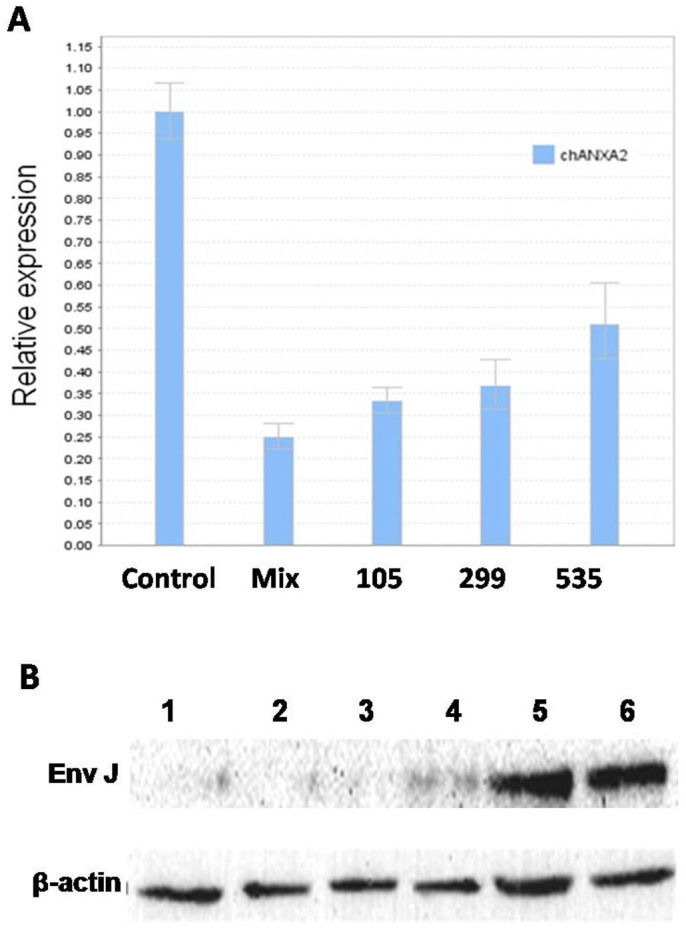
(Qin) siRNA against chANXA2 in DF1 cells DF1 cells were transfected with siRNA (50 pmol) against chANXA2 for 6 hr, and then the cells were infected with ALV-J at an MOI of 1 for 72 hr. (A), The siRNA effects on chANXA2 was detected with real-time PCR; (B), The infection/replication of ALV-J was analyzed with western blot. Lane 1, 2, 3 and 4, DF1 cells transfected with siRNA Mix (co-transfected with 535, 105 and 299), 535, 105 and 299 against chANXA2 respectively; lane5, DF1 cells transfected with control siRNA; lane6, DF1 cells with Mock.

To further evaluate whether the low expression of chANXA2 could confer resistance to ALV-J infection in its susceptible cells, we did siRNA against chANXA2 in DF1 cell, and then tested the effect on the ALV-J infection/replication. As described in Fig. 3A, real-time PCR showed that all four siRNA against chANXA2 tested could efficiently reduce chANXA2 mRNA level. And the western blot showed that all these four siRNA against chANXA2 tested could significantly decrease the infection/replication of ALV-J in DF1 cells (Fig. 3B).

### Over-expression of chANXA2 permitting the entry of ALV-J into its non-permissible cells

**Figure 4:**
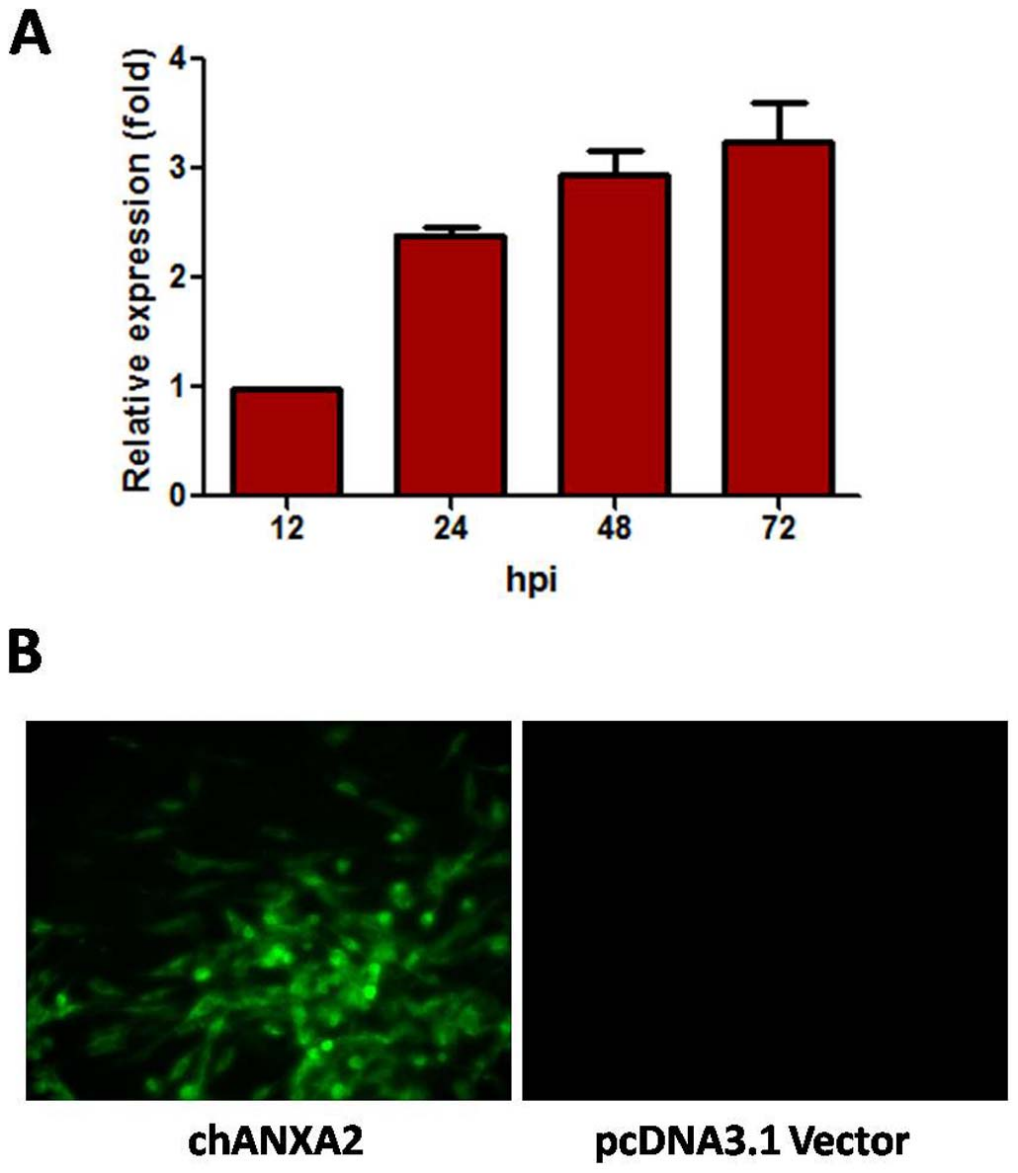
(Qin) Infection of the chANXA2-transfected cells. Geese embryo fibroblasts (GEF) and 293T cells were transfected with chANXA2, and the transfected cells were then infected with ALV-J at an MOI of 5. (A), The replication of ALV-J in the 293T cells transfected with ANXA2 was measured by real-time PCR; (B), The replication of ALV-J in the GEF cells transfected with ANXA2 was measured IFA after inoculating the homogenate from the transfected GEF cells to the DF1 cells.

To extend our finding and investigate whether the over-expression of the chANXA2 protein in ALV-J non-permissible cells could induce susceptibility to ALV-J infection, the replication of ALV-J in the 293T cells transfected with chANXA2 was analysed by IFA and real-time PCR. Real-time PCR revealed that the relative expression of the ALV-J envelope gene was increased in the chANXA2-transfected 293T cells at 72 h post-infection (Fig. 4A). However, the 293T cells that were transfected with chANXA2 exhibited no visible specific fluorescence with the JE9 antibody to ALV-J Env. These data indicate that ALV-J could enter the 293T cells that were over-expressing chANXA2, but the viral replication of the ALV-J was restricted in the transfected 293T cells. To further confirm this finding, we also transfected chANXA2 into goose embryo fibroblast (GEF) cells in which the ALV-J could not grow. After 48 h, the transfected GEFs were infected with ALV-J. The GEF cells were maintained in DMEM medium for 2 days. Next, the mixture of GEF cells and supernatants were inoculated into fresh DF1 cells. Interestingly, as shown in Fig. 4B, virus was recovered from the GEF cells transfected with chANXA2, but no virus was obtained from the GEF cells that were transfected with the control plasmid. Together, these data demonstrate that the over-expression of chANXA2 in 293T or GEF cells can support ALV-J entry into these ALV-J non-permissible cells.

## Discussion

Viral infection and pathogenesis are initiated by the binding of the viral surface protein to its cellular receptor. And the identification of viral cell receptor is critical not only for better understanding the molecular events for viral infection, but also for developing novel anti-viral strategies. The traditional methods for identifying viral receptors mainly depend on the expression and purification of the viral binding proteins^11^,^12^,^13^,^14^. In this study, we reported the first isolation and identification of chANXA2 as a novel functional receptor for ALV-J entry by using an ALV-J-resistant cell line without the procedure of viral protein purification. And the isolation of chANXA2 as ALV-J Env binding protein was further confirmed in this study by using fusion protein SUJ-rIgGFc, which had used in the traditional method for ALV receptor isolation. And the interaction between ALV-J Env and chANXA2 was confirmed using Co-IP. Our findings highlight that virus-resistant cell lines that are constructed through the expression of viral receptor-binding protein in susceptible cells can be widely used as an efficient tool for the isolation of functional receptors for viral entry and novel anti-viral targets.

The blocking of chANXA2 with a specific antibody and siRNA against chANXA2 can both effectively inhibit the infection/replication of ALV-J suggests chANXA2 can be as a novel host marker for anti-ALV-J strategies and as a new model system for examining the molecular events of retroviral entry into the host. To our knowledge, this is the first demonstration of the inhibition of ALV-J infection by antibodies against one of the host's protein, which highlights the fact that chANXA2 can serve as a novel functional receptor for ALV-J entry and provide a host marker for anti-ALV-J strategies. In 2006, chNHE1 was identified by Ning et al as a receptor for ALV-J infection^14^, and Kucerova et al also recently demonstrated that a single amino-acid substitution of W38 in chNHE1 abrogates ALV-J infection^21^. However, no data have yet shown that antibodies to chNHE1 can block or inhibit ALV-J infection so far. In present study, we did not rule out the fact that chNHE1 is a receptor for ALV-J infection, but we did identify chANXA2 as a novel receptor for ALV-J infection. However, the potential interaction between chNHE1 and chANXA2 need to be investigated. Either chNHE1 requires chANXA2 or chANXA2 requires chNHE1 when they as ALV-J receptors need to be further tested. The over-expression of chANXA2 could permit the entry of ALV-J into ALV-J non-permissible cells further supports the conclusion of chANXA2 as a novel functional receptor for ALV-J infection.

ANXA2 is a member of the annexin family and plays vital roles in regulating cellular functions including the endo- and exocytotic pathways, the generation of plasmin and calcium-dependent F-actin filament bundling^20^,^22^,^23^,^24^. It has been reported that ANXA2 can link HCMV to a phospholipid membrane and enhance virus-membrane fusion and is a receptor for respiratory syncytial virus^25^,^26^. More recently, ANXA2 was also reported to play critical roles in the viral entry/replication of human papillomavirus type 16, enterovirus 71 and hepatitis C virus^27^,^28^,^29^,^30^. Furthermore, ANXA2 plays a cell type-dependent role in regulating HIV infectivity^29^. These previous reports regarding ANXA2 indicate that, because ALV-J Env binding protein was isolated using an ALV-J resistant cell line in the present study, ANXA2 might also play vital roles in ALV-J infection. The roles of ANXA2 as viral receptor or in viral infectivity reported may relate with its endo- and exocytotic pathways. However, the mechanism of these novel functions of ANXA2 need to be further elucidated. Further studies will also shed light on the roles of chANXA2 in myelomas and haemangiomas induced by ALV-J infection^31^.

## Methods

### Cells and viruses

HEK293T cells were maintained in Dulbecco's modified Eagle's medium (DMEM) supplemented with 10% fetal bovine serum (FBS) and antibiotics. Geese embryo fibroblast (GEF), DF-1 and pcDNA-env_DF1 cells^18^ were maintained in DMEM supplemented with 5% fetal bovine serum and antibiotics. The ALV-J strain (JS09GY07) was isolated from layer chickens with both hemangioma and myeloid leukosis. And the virus was titrated in DF1 cells to determine the TCID_50_ by the Reed and Muench method^32^.

### Construction and purification of fusion protein SUJ-rIgGFc

The gp85 coding region including the signal peptide was amplified by PCR from the isolate JS09GY07. And the IgGFc heavy chain of rabbit was obtained with RT-PCR from the RNA of rabbit blood cells. The SU fragment and the IgGFc heavy chain were ligated with BamHI restriction enzyme site and the fusion fragment was cloned into the eukaryotic expression vector pcDNA3.1 for sequencing. After that, we cloned SUJ-IgGFc into adenovirus vector for expressing fusion protein in adenovirus expression system according to the manufacture's instruction. As expected, a recombinant adenovirus plasmid pAD-SUJ-rIgGFc containing a 1.8-kilobase (kb) fragment which fused ALV-J SU gene and the Fc region of rabbit IgG was constructed, and was transfected into 293T cell to generate recombinant adenovirus rAd-SUJ-rIgGFc which expressed fusion protein SUJ-rIgGFc, and it was purified with protein A agarose for subsequent protein precipitation.

### Preparation of membrane extracts

pcDNA-env_DF1 cells and DF1 cells in 100 mm dishes were harvested by scraping with a rubber policeman and homogenised with NP-40 lysis buffer containing 25 mM Tris, 150 mM NaCl, 1 mM EDTA, 1% NP-40, 5% glycerol (pH7.4) and a protease inhibitor cocktail (Roche). Intact cells and nuclei in the resulting extract were sedimented by centrifugation at 4°C for 5 min at 6000 g. The membrane proteins in the supernatant were sedimented by an additional spin at 13200 g for 1 h at 4°C and resuspended in 1% NP-40 lysis buffer. All extracts were stored at −80°C until use.

### Immunoprecipitation and mass spectrometry

The membrane proteins from the pcDNA-env_DF1 cells were immunoprecipitated with the monoclonal antibody JE9, which is specific to ALV-J Env and Resin A (Thermo Scientific). The membrane proteins from the DF1 cells were precipitated with the purified protein SUJ-rIgGFc and protein A agarose (Beyotime, China). Precipitated proteins were separated by SDS-PAGE, stained with a Silver Stain Kit (Thermo Scientific) and analysed with mass spectrometry. In Co-immunoprecipitation (Co-IP), 293T cells were co-transfected with plasmid pcDNA3.1-EnvJ, and chANXA2 for 48 h, and then lysed in lysis buffer. The cell lysates were immunoprecipitated with mAb JE9 at 4°C overnight, and then incubated with protein A agarose for 1 hr. After three washes in lysis buffer, the lysates were analyzed using JE9 and antibody against chANXA2 in western blot.

### Antibody blocking assay

The DF1 cells were pre-treated with different concentrations of anti-ANXA2 (C-16, Santa Cruz) for 2 h and then the cells were infected with ALV-J or ALV-A at an MOI of 5. The cells were maintained in DMEM with 1% fetal bovine serum and different concentrations of antibodies. Normal goat-IgG was used as the isotype control antibody in the blocking assay. The replication of ALV-J in the treated cells was analysed with IFA, real-time PCR, TCID_50_ and western blot.

### siRNA against chANXA2 in DF1 cells

Three siRNA (535, 105 and 299) against chANXA2 were synthesized (Invitrogen). The sequences of these siRNA were listed in Table 1. DF1 cells were transfected with siRNA (50 pmol) against chANXA2 or control siRNA for 6 hours, and then the cells were infected with ALV-J at a MOI of 1 for 72 h. The siRNA effects on chANXA2 was detected with Real-time PCR, and the replication of ALV-J in DF1 cells transfected with siRNA was analyzed through western blot using mAb JE9. The primers of Real-time PCR for chANXA2 were listed in Table 1.

### Infection of chANXA2-transfected cells

293T or geese embryo fibroblasts (GEF) cells were transfected with chANXA2 for 48 h. Then, the transfected cells were infected with ALV-J at an MOI of 5 (5 TCID_50_/cell based on the titer obtained from DF1 cells) for 2 h followed by three washes with PBS and treatment with acid glycine (pH 3.0) for 1 min. After further three washes with PBS, the 293T and GEF cells were maintained in DMEM with 1% FBS for 48 h. For the 293T cells, the replication of ALV-J was analysed with IFA and real-time PCR at 12 h, 24 h, 48 h and 72 h post-infection. For the GEF cells, the cells and supernatants were homogenised and inoculated into the DF1 cells, and viral replication in the DF1 cells was analysed with IFA with JE9 at day 7 post-inoculation.

### Real-time PCR

RNA was isolated from the cell lysates using the Total RNA Miniprep Kit (Axygen) according to the manufacturer's instructions. The RNA was reverse-transcribed using PrimerScript RT Reagent Kit (Takara) with primers that are specific to the ALV-J gp37 and ANXA2 gene according to the manufacturer's instruction. Chicken 18S and human β-actin were used as an internal control in this assay. The primers of Real-time PCR for ALV-J gp37, ANXA2 and chicken 18S and human β-actin were listed in Table 1.

### Indirect immunofluorescence assay (IFA) and western blot analysis

Indirect immunofluorescence assays (IFAs) were performed on the 293T and DF1 cells. The monoclonal antibody JE9, which is specific to the Env of ALV-J, was used as the primary antibody^19^. FITC-goat anti-mouse IgG was used as the secondary antibody. Western blot analyses were performed on cell lysates. JE9 or anti-β-actin or anti-chANXA2 antibody was used as the primary antibody, and HRP-conjugated goat anti-mouse or HRP-conjugated donkey anti-goat was used as the secondary antibody (Santa Cruz).

## Author Contributions

M.M., A.J.Q., K.Q. and J.Q.Y. conceived and designed the experiments; M.M. and L.W. performed the experiments; M.M., A.J.Q., K.Q., X.M.H., H.X.S. and J.Q.Y. analyzed the data; M.M., L.W., X.M.H., H.X.S. and J.Q.Y. contributed reagents/materials/analysis tools; M.M., A.J.Q., J.Q.Y. and K.Q. contributed to the writing of the manuscript. M.M., L.W. and J.Q.Y. prepared figures. All authors reviewed the manuscript.

## Figures and Tables

**Table 1:** Sequences of primers for Real-time PCR and siRNA against chANXA2

	Name	Sequence (5′ to 3′)
RT-PCR	ALV-J gp37	Forward: TGCGTGCGTGGTATTATTTC
		Reverse: AATGGTGAGGTCGCTGACTGT
	chANXA2	Forward: ATCAACATCCTGACAAACCG
		Reverse: TAAGTGCTGCAGAAAGTTCC
	Chicken 18S	Forward: TCAGATACCGTCGTAGTTCC
		Reverse: TTCCGTCAATTCCTTTAAGTT
	Human β-actin	Forward: CACGAAACTACCTTCAACTCC
		Reverse: CATACTCCTGCTTGCTGATC
siRNA	105	Forward: UAACUGUGGCAUAUGCACUUGGAGG
		Reverse: CCUCCAAGUGCAUAUGCCACAGUUA
	299	Forward: UGCAGCACUUAAGUCUGCUCUGUCA
		Reverse: UGACAGAGCAGACUUAAGUGCUGCA
	535	Forward: CAUCUGGUGACUUCCGCAAGCUAAU
		Reverse: AUUAGCUUGCGGAAGUCACCAGAUG

## References

[b1] Mothes W., Boerger A. L., Narayan S., Cunningham J. M. & Young J. A. Retroviral entry mediated by receptor priming and low pH triggering of an envelope glycoprotein. Cell 103, 679–689 (2000). 11106737 10.1016/s0092-8674(00)00170-7

[b2] Lindemann D., Steffen I. & Pohlmann S. Cellular entry of retroviruses. Adv. Exp. Med. Biol. 790, 128–149 (2013). 23884589 10.1007/978-1-4614-7651-1_7

[b3] Dimitrov D. S. Virus entry: molecular mechanisms and biomedical applications. Nat. Rev. Microbiol. 2, 109–122 (2004). 15043007 10.1038/nrmicro817 PMC7097642

[b4] Tran K. *et al.* Vaccine-elicited primate antibodies use a distinct approach to the HIV-1 primary receptor binding site informing vaccine redesign. Proc. Natl Acad. Sci. USA 111, E738–747 (2014). 24550318 10.1073/pnas.1319512111 PMC3932900

[b5] Bogers W. M. *et al.* A novel HIV-CCR5 receptor vaccine strategy in the control of mucosal SIV/HIV infection. Aids 18, 25–36 (2004). 15090826 10.1097/00002030-200401020-00003

[b6] Martin K. A. *et al.* CD4-independent binding of SIV gp120 to rhesus CCR5. Science 278, 1470–1473 (1997). 9367961 10.1126/science.278.5342.1470

[b7] Hutter G. *et al.* Long-term control of HIV by CCR5 Delta32/Delta32 stem-cell transplantation. N. Engl. J. Med. 360, 692–698 (2009). 19213682 10.1056/NEJMoa0802905

[b8] Olofsson S. & Bergstrom T. Glycoconjugate glycans as viral receptors. Ann. Med. 37, 154–172 (2005). 16019714 10.1080/07853890510007340

[b9] Adedeji A. O. *et al.* Novel Inhibitors of Severe Acute Respiratory Syndrome Coronavirus Entry That Act by Three Distinct Mechanisms. J. Virol. 87, 8017–8028 (2013). 23678171 10.1128/JVI.00998-13 PMC3700180

[b10] Dimitrov D. S. The secret life of ACE2 as a receptor for the SARS virus. Cell 115, 652–653 (2003). 14675530 10.1016/S0092-8674(03)00976-0 PMC7133233

[b11] Bates P., Young J. A. & Varmus H. E. A receptor for subgroup A Rous sarcoma virus is related to the low density lipoprotein receptor. Cell 74, 1043–1051 (1993). 8402880 10.1016/0092-8674(93)90726-7

[b12] Brojatsch J., Naughton J., Rolls M. M., Zingler K. & Young J. A. CAR1, a TNFR-related protein, is a cellular receptor for cytopathic avian leukosis-sarcoma viruses and mediates apoptosis. Cell 87, 845–855 (1996). 8945512 10.1016/s0092-8674(00)81992-3

[b13] Adkins H. B. *et al.* Identification of a cellular receptor for subgroup E avian leukosis virus. Proc. Natl Acad. Sci. USA 94, 11617–11622 (1997). 9326659 10.1073/pnas.94.21.11617 PMC23555

[b14] Chai N. & Bates P. Na+/H+ exchanger type 1 is a receptor for pathogenic subgroup J avian leukosis virus. Proc. Natl Acad. Sci. USA 103, 5531–5536 (2006). 16567631 10.1073/pnas.0509785103 PMC1459389

[b15] Elleder D. *et al.* The receptor for the subgroup C avian sarcoma and leukosis viruses, Tvc, is related to mammalian butyrophilins, members of the immunoglobulin superfamily. J. Virol. 79, 10408–10419 (2005). 16051833 10.1128/JVI.79.16.10408-10419.2005 PMC1182627

[b16] Crittenden L. B. & Salter D. W. A transgene, alv6, that expresses the envelope of subgroup A avian leukosis virus reduces the rate of congenital transmission of a field strain of avian leukosis virus. Poultry. Sci. 71, 799–806 (1992). 1319049 10.3382/ps.0710799

[b17] Hunt H. D., Lee L. F., Foster D., Silva R. F. & Fadly A. M. A genetically engineered cell line resistant to subgroup J avian leukosis virus infection (C/J). Virology 264, 205–210 (1999). 10544146 10.1006/viro.1999.9993

[b18] Ye J. Q. A., Shao H., Liu H., Jin W. & Liu Y. Development of chicken embryo fibroblast cell line resistant to J subgroup avian leukosis virus (ALV-J) infection. Bing. Du. Xue. Bao. 21, 456–460 (2005).

[b19] Qin A., Lee L. F., Fadly A., Hunt H. & Cui Z. Development and characterization of monoclonal antibodies to subgroup J avian leukosis virus. Avian. Dis. 45, 938–945 (2001). 11785897

[b20] Okuse K. *et al.* Annexin II light chain regulates sensory neuron-specific sodium channel expression. Nature 417, 653–656 (2002). 12050667 10.1038/nature00781

[b21] Kucerova D. *et al.* Nonconserved tryptophan 38 of the cell surface receptor for subgroup J avian leukosis virus discriminates sensitive from resistant avian species. J. Virol. 87, 8399–8407 (2013). 23698309 10.1128/JVI.03180-12 PMC3719790

[b22] Donato R. & Russo-Marie F. The annexins: structure and functions. Cell calcium 26, 85–89 (1999). 10598271 10.1054/ceca.1999.0079

[b23] Gerke V. & Moss S. E. Annexins: from structure to function. Physiol. Rev. 82, 331–371 (2002). 11917092 10.1152/physrev.00030.2001

[b24] Kim J. & Hajjar K. A. Annexin II: a plasminogen-plasminogen activator co-receptor. Front. Biosci. 7, d341–348 (2002). 11815288 10.2741/kim

[b25] Raynor C. M., Wright J. F., Waisman D. M. & Pryzdial E. L. Annexin II enhances cytomegalovirus binding and fusion to phospholipid membranes. Biochemistry 38, 5089–5095 (1999). 10213612 10.1021/bi982095b

[b26] Malhotra R. *et al.* Isolation and characterisation of potential respiratory syncytial virus receptor(s) on epithelial cells. Microbes. Infect. 5, 123–133 (2003). 12650770 10.1016/s1286-4579(02)00079-5

[b27] Dziduszko A. & Ozbun M. A. Annexin A2 and S100A10 regulate human papillomavirus type 16 entry and intracellular trafficking in human keratinocytes. J. Virol. 87, 7502–7515 (2013). 23637395 10.1128/JVI.00519-13 PMC3700289

[b28] Yang S. L., Chou Y. T., Wu C. N. & Ho M. S. Annexin II binds to capsid protein VP1 of enterovirus 71 and enhances viral infectivity. J. Virol. 85, 11809–11820 (2011). 21900167 10.1128/JVI.00297-11 PMC3209289

[b29] Rai T., Mosoian A. & Resh M. D. Annexin 2 is not required for human immunodeficiency virus type 1 particle production but plays a cell type-dependent role in regulating infectivity. J. Virol. 84, 9783–9792 (2010). 20631122 10.1128/JVI.01584-09 PMC2937750

[b30] Backes P. *et al.* Role of annexin A2 in the production of infectious hepatitis C virus particles. J. Virol. 84, 5775–5789 (2010). 20335258 10.1128/JVI.02343-09 PMC2876593

[b31] Payne L. N. & Nair V. The long view: 40 years of avian leukosis research. Avian. Pathol. 41, 11–19 (2012). 22845317 10.1080/03079457.2011.646237

[b32] Reed L. J. & Muench H. A simple method for estimating 50 percent endpoints. Am. J. Hyg. 37, 493 (1938).

